# Small high-density lipoprotein is associated with monocyte subsets in stable coronary artery disease

**DOI:** 10.1016/j.atherosclerosis.2014.10.015

**Published:** 2014-12

**Authors:** Konstantin A. Krychtiuk, Stefan P. Kastl, Stefan Pfaffenberger, Thomas Pongratz, Sebastian L. Hofbauer, Anna Wonnerth, Katharina M. Katsaros, Georg Goliasch, Ludovit Gaspar, Kurt Huber, Gerald Maurer, Elisabeth Dostal, Stanislav Oravec, Johann Wojta, Walter S. Speidl

**Affiliations:** a Department of Internal Medicine II – Division of Cardiology, Medical University of Vienna, Vienna, Austria; b Ludwig Boltzmann Cluster for Cardiovascular Research, Vienna, Austria; c Krankenanstalten Dr. Dostal, Vienna, Austria; d 2nd Department of Internal Medicine, Faculty of Medicine, Comenius University, Bratislava, Slovakia; e 3rd Medical Department, Wilhelminenhospital, Vienna, Austria; f Core Facilities, Medical University of Vienna, Vienna, Austria

**Keywords:** HDL, Small HDL, Atherosclerosis, Monocytes, Monocyte subsets, Inflammation

## Abstract

**Objective**: High-density lipoprotein (HDL) particles are heterogeneous in structure and function and the role of HDL subfractions in atherogenesis is not well understood. It has been suggested that small HDL may be dysfunctional in patients with coronary artery disease (CAD). Monocytes are considered to play a key role in atherosclerotic diseases. Circulating monocytes can be divided into three subtypes according to their surface expression of CD14 and CD16. Our aim was to examine whether monocyte subsets are associated with HDL subfractions in patients with atherosclerosis. **Methods**: We included 90 patients with angiographically stable CAD. Monocyte subsets were defined as classical monocytes (CD14++CD16-; CM), intermediate monocytes (CD14++CD16+; IM) and non-classical monocytes (CD14+CD16++; NCM). HDL subfractions were measured by electrophoresis on polyacrylamide gel. **Results**: Serum levels of small HDL correlated with circulating pro-inflammatory NCM and showed an inverse relationship to circulating CM independently from other lipid parameters, risk factors, inflammatory parameters or statin treatment regime, respectively. IM were not associated with small HDL. In particular, patients with small HDL levels in the highest tertile showed dramatically increased levels of NCM (14.7 ± 7% vs. 10.7 ± 5% and 10.8 ± 5%; *p* = 0.006) and a decreased proportion of CM (79.3 ± 7% vs. 83.7 ± 6% and 83.9 ± 6%; *p* = 0.004) compared to patients in the two lower tertiles. In contrast, intermediate HDL, large HDL and total HDL were not associated with monocyte subset distribution. **Conclusion**: Small HDL levels are associated with pro-inflammatory NCM and inversely correlated with CM. This may suggest that small HDL could have dysfunctional anti-inflammatory properties in patients with established CAD.

## Highlights

• Small HDL levels are associated with non-classical monocytes in stable CAD.
• Classical monocytes are inversely associated with small HDL levels.
• Associations are independent of other lipid parameters, risk factors, inflammatory parameters or statin treatment regime.
• Inflammatory markers do not vary according to small HDL levels.

## 1 Introduction

Despite major advances in the field of vascular medicine, cardiovascular diseases remain the leading cause of death in the western world [1]. Large clinical and epidemiological studies have consistently and extensively demonstrated a strong inverse relationship between levels of high density lipoprotein (HDL) and cardiovascular diseases, independently of low-density lipoprotein levels (LDL) [2], [3]. Consequently, the concept of raising HDL levels by therapeutic means became a strong research focus. However, contrary to expectations, increasing HDL levels pharmacologically failed to translate to lower one's cardiovascular risk so far [4]. One of many prominent examples was torcetrapib, an inhibitor of the cholesterylester transfer protein (CETP). Despite a strong increase in HDL levels, mortality and morbidity in patients receiving the drug was increased causing a premature termination of a total of six studies, including the ILLUMINATE study [5]. A growing body of evidence suggests that under certain pathological conditions, HDL function might be impaired, evidencing the importance of HDL functionality [6], [7].

HDLs are a heterogeneous class of lipoprotein particles with varying structure, metabolism and anti-atherogenic properties [8], [9]. Amongst HDL subgroups, small HDL from healthy subjects has been shown to exert potent atheroprotective effects including increased potential of cholesterol efflux, and stronger anti-oxidative as well as anti-inflammatory activities [9]. However, under conditions such as atherogenic dyslipidemia, these properties were shown to be compromised [10], [11]. In clinical studies, small HDL was associated with presence and severity of atherosclerotic disease, while large HDL was negatively correlated with presence of coronary artery disease (CAD) and severity and progression of the disease [12], [13], [14].

Atherosclerosis is considered to be an inflammatory disease in which monocytes play a key role in all stages of the disease [15]. Within the last decade, monocyte heterogeneity was acknowledged to play a role in atherogenesis [16], [17]. Mononuclear cells can be distinguished into at least three subsets by their surface expression pattern of CD14 and CD16, namely into classical monocytes (CD14++CD16−; CM), intermediate monocytes (CD14++CD16+; IM) and non-classical monocytes (CD14+CD16++; NCM), the latter two being viewed as pro-inflammatory cells [18], [19], [20]. The proportion of these pro-inflammatory subgroups was shown to be extended in patients with CAD, correlated with intima media thickness in healthy adults and was associated with plaque stability in stable and unstable patients [21], [22], [23], [24]. Total cholesterol, LDL-cholesterol and triglycerides were correlated with the pro-inflammatory subset NCM, while HDL-cholesterol showed a negative association [25], [26]. In more than 900 patients with stable CAD, IM were predictive of cardiovascular events [27].

The functional consequence of otherwise anti-atherogenic small HDL in dyslipidemia, metabolic syndrome and atherosclerotic disease is unknown [28], [29]. As inflammation is crucially involved in most of these conditions, the aim of our study was to examine whether the different HDL subfractions are associated with the innate immune status evidenced by monocyte subset distribution.

## 2 Materials and methods

### 2.1 Subjects and study design

In this single-center, cross-sectional study, we included 90 consecutive patients undergoing elective coronary angiography due to stable coronary artery disease between September 2009 and April 2010. Patients gave written, informed consent for this study, which was approved by the local ethical committee (Medical University of Vienna, EK Nr. 020/2008) and complies with the Declaration of Helsinki. Inclusion criteria comprised male and female patients aged >18 years with stable CAD undergoing elective coronary angiography. Excluded were patients with a recent acute coronary syndrome defined as ST-elevating myocardial infarction (STEMI), non-STEMI or unstable angina with or without percutaneous coronary intervention (PCI) within the last three months, patients with heart failure, valvular disease, malignant disease, liver, kidney or other acute and chronic inflammatory diseases. Arterial hypertension was defined as systolic blood pressure ≥140 mmHg, diastolic blood pressure ≥90 mmHg in at least two measurements or the current use of antihypertensive drugs. Subjects were defined as being diabetic if treated for insulin or non-insulin-dependent diabetes mellitus or having a plasma fasting glucose ≥126 mg/dL in at least two measurements. Extent of coronary artery disease is given as the number of epicardial coronary arteries with a ≥70% stenosis. High-dose statin treatment was defined as treatment with atorvastatin with a dosage of at least 40 mg or rosuvastatin at a dosage of at least 10 mg daily.

### 2.2 Blood sampling

Blood was drawn in the morning prior to elective coronary angiography after venipuncture from an antecubital vein using a 21-gauge butterfly needle (0.8 mm × 19 mm; Greiner Bio-One, Kremsmünster, Austria). After the initial 3 mL of blood were discarded, blood was drawn into an EDTA tube (Greiner Bio-One) for immediate analysis by flow cytometry. Furthermore, a 3.8% sodium citrate Vacuette tube (Greiner Bio-One; nine parts of whole blood, one part of sodium citrate 0.129 M/L), a serum separator tube (Greiner Bio-One) and an EDTA tube (Greiner Bio-One) were collected, immediately centrifuged (4 °C; 3000RPM for 15 min) and stored at −80 °C for later analysis.

### 2.3 Laboratory measurements

Granulocyte colony-stimulating factor (G-CSF), macrophage colony-stimulating factor (M-CSF) and interleukin-6 (IL-6) were measured using a specific enzyme-linked immunosorbent assay (ELISA; R&D Systems, Minneapolis, MN, USA), while plasma levels of interleukin-10 (IL-10) and granulocyte–macrophage colony-stimulating factor (GM-CSF) were quantified using a customized multiplex assay (Luminex Assay, R&D Systems, catalog number FCST03). For the detection of standard laboratory markers including high-sensitive C-reactive protein (hsCRP), blood was analyzed in the central laboratory of the General Hospital of Vienna.

### 2.4 Flow cytometry

**Fig. 1:**
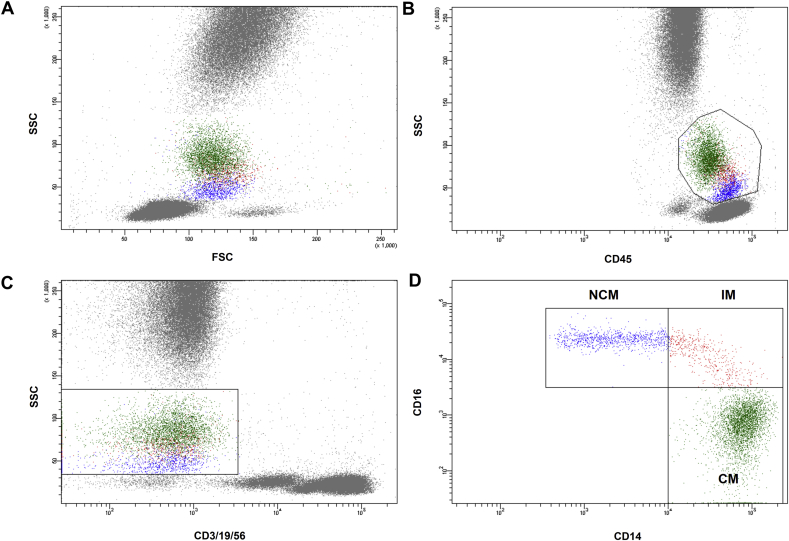
Gating strategy used for monocyte subset discrimination. Monocytes were defined as CD45 positive cells (B) exhibiting a typical forward (FSC) and sideward scatter (SSC) profile (A). To exclude possible contamination with T-cells, B-cells and Natural Killer cells, cells that stained for CD3, CD19 and CD56 were excluded, respectively (C). Remaining CD45+CD3/19/56− cells with a typical FSC/SSC profile were considered monocytes and distinguished according to their CD14 and CD16 surface expression into classical monocytes (CD14++CD16−), intermediate monocytes (CD14++CD16+) and non-classical monocytes (CD14+CD16++) (D).

Whole blood flow cytometry for determination of leukocyte and monocyte subset distribution was performed using a FACS Canto II with the FACS Diva Software (both Becton Dickinson). The staining and gating strategy is outlined in Fig. 1. Briefly, 100 μL of EDTA-anticoagulated whole blood was stained with saturating concentrations of the following fluorochrome-conjugated monoclonal antibodies (mAbs): Peridinin chlorophyll protein (PerCP)-labeled mAb for CD45 (Beckton Dickinson, catalog number 345809), fluorescein isothiocyanate (FITC)-labeled mAb for CD14 (Beckton Dickinson, catalog number 345784), allophycocyanin (APC)-H7-labeled mAb for CD16 (Beckton Dickinson, catalog number 560195), APC-labeled mAb for CD3 (Beckton Dickinson, catalog number 345767), CD19 (Beckton Dickinson, catalog number 345791) and CD56 (Beckton Dickinson, catalog number 341027) and corresponding isotype controls. After incubation for 15 min in the dark, 1.5 mL lysing solution (BD FACS lysing solution BD Biosciences) was added. After an additional 15 min of incubation in darkness, cells were washed three times by adding 1 mL PBS and centrifugation at 820 RPM for 5 min each. Cells were then resuspended in 1 mL fixative solution (FACS Flow, reagent-grade water and BD Cellfix™) for FACS-analysis. Monocytes were identified as CD45-positive and CD3-, CD19-and CD56-negative cells exhibiting a specific forward and sideward scatter profile. Individual monocyte subsets were defined according to a recently published international consensus document as “classical monocytes” (CM; CD14++CD16-), “intermediate monocytes” (IM; CD14++CD16+) and “non-classical monocytes” (NCM; CD14+CD16++) [19]. Absolute numbers of monocytes were calculated using leukocyte count as determined by the central laboratory and counts of CD45+ cells as determined by flow cytometry. For all calculations relative numbers of monocyte subsets in percentage of total monocytes were used. The coefficient of variation for relative monocyte count was 4.9%.

### 2.5 Lipid measurements

For all lipid measurements, only previously unthawed serum samples were used. Levels of total cholesterol, HDL, LDL and triglycerides were measured by the general laboratory of *Krankenanstalten Dr. Dostal* using enzymatic methods. For quantification of HDL subfractions, the Quantimetrix HDL Lipoprint System^®^ (Quantimetrix Corporation, Redondo Beach, CA, USA) was used according to manufacturer's instructions as previously described [30]. Briefly, HDL subfraction separation and quantification with this system is based upon high resolution polyacrylamide gel electrophoresis and divides HDL into 10 subfractions. Subfraction 1–3 represent large HDL particles, subset 4–7 the intermediate HDL subtype while subsets 8–10 represent small HDL particles [31].

### 2.6 Statistical analysis

Categorical variables are expressed as counts or percentages and were compared by the *χ*^2^ or by Fisher's exact test where appropriate. Continuous variables are given as mean ± standard deviation. Parametric data was compared using ANOVA, while skewed data (assessed by the Kolmogorov–Smirnov test) was compared by ANOVA after log-transformation. Correlations were calculated using Pearson's correlation coefficient. Linear regression models were calculated for all three monocyte subsets, respectively. Clinical characteristics, statin treatment or lipid parameters were added to the models when they were associated with monocyte subsets or small HDL levels by a *p*-value <0.2. In addition, BMI was added to the model as it has been shown to modify the association between lipid levels and monocyte subsets. A value of *p* < 0.05 (two-tailed) was considered statistically significant. All statistical analyses were performed with the Predictive Analysis SoftWare PASW Statistics 18.0 (IBM, Armonk, NY, USA).

## 3 Results

### 3.1 Patient characteristics

Ninety patients with angiographically proven stable coronary artery disease were enrolled in this study. Table 1 shows the clinical characteristics. Mean age was 64.1 ± 10.0 years, 72 patients (80%) were male and 21 (23%) were smokers. 25 (28%) had single vessel disease, 36 (40%) had two diseased coronary arteries and 29 patients (32%) had triple vessel disease. 31% of patients were on a high-dose statin, 52% of patients received low-dose statin treatment while 17% of patients were not treated with statins.

**Table 1:** Clinical characteristics of the study population.

	Total *n* = 90	Small HDL tertile 1 *n* = 26	Small HDL tertile 2 *n* = 27	Small HDL tertile 3 *n* = 37	*p*-value
Range, mg/dL	2–20	2–8	9–12	13–20	
Age (years)	64.1 ± 10.0	64 ± 10.6	67.0 ± 9.2	62.0 ± 8.2	0.11
Male gender, *n* (%)	72 (80)	22 (84)	21 (78)	29 (78)	0.78
Hypertension, *n* (%)	80 (89)	23 (89)	23 (85)	34 (92)	0.70
Diabetes Mellitus, *n* (%)	27 (30)	5 (19)	10 (37)	12 (32)	0.34
Current smoker, *n* (%)	21 (23.3)	9 (35)	6 (22)	6 (16)	0.23
CAD Extent (VD)					0.70
1VD, *n* (%)	25 (28)	9 (35)	6 (22)	10 (27)	
2VD, *n* (%)	36 (40)	16 (31)	11 (41)	9 (46)	
3VD, *n* (%)	29 (32)	9 (35)	10 (37)	10 (27)	
Statin Treatment					0.89
No Statin, *n* (%)	15 (17)	4 (15)	6 (22)	5 (14)	
Low–dose Statin, *n* (%)	47 (52)	13 (50)	14 (52)	20 (54)	
High–dose Statin, *n* (%)	28 (31)	9 (35)	7 (26)	12 (32)	
BMI (kg/m^2^)	29 ± 4.7	28.2 ± 5.2	28.8 ± 4.2	29.8 ± 4.6	0.38
HbA1c (%)	6.1 ± 0.9	6.2 ± 1.0	5.9 ± 0.9	6.2 ± 0.8	0.65
Creatinine (mg/dL)	1.1 ± 0.3	1.0 ± 0.3	1.1 ± 0.2	1.1 ± 0.3	0.51
Leukocytes (G/L)	7.1 ± 1.7	6.9 ± 1.6	7.1 ± 1.8	7.2 ± 1.8	0.90
Triglycerides (mg/dL)	153.3 ± 81.2	124.5 ± 47.3	132.2 ± 61.8	188.9 ± 98.8	<0.001
Total cholesterol (mg/dL)	164.6 ± 39	147.5 ± 40.1	156.4 ± 34.9	182.5 ± 33.9	<0.001
HDL (mg/dL)	40.1 ± 13.4	38.8 ± 13.3	43.3 ± 14.5	40.5 ± 12.7	0.47
VLDL (mg/dL)	28.6 ± 9.4	24.7 ± 7.7	25.3 ± 6.6	40.5 ± 12.7	<0.001
LDL (mg/dL)	93.3 ± 30.8	84.5 ± 32.3	86.6 ± 28.0	104.2 ± 29.0	0.016

BMI body mass index; HDL high density lipoprotein; VLDL very low density lipoprotein; LDL low density lipoprotein; CAD coronary artery disease; VD vessel disease; Statin dose: High-dose statin treatment was defined as treatment with atorvastatin with a dosage of at least 40 mg or rosuvastatin at a dosage of at least 10 mg daily.

### 3.2 Correlation of HDL subfractions with lipid parameters and cardiovascular risk factors

Small HDL levels were not associated with total HDL levels but showed significant correlations with triglycerides, VLDL, LDL and total cholesterol levels (Table 2). In contrast, large HDL that was inversely correlated with small HDL, highly correlated with total HDL and correlated inversely with VLDL and triglyceride levels. Intermediate HDL correlated with large HDL but showed in addition an association with LDL and VLDL. In contrast to small HDL, intermediate HDL did not correlate with triglycerides. Intermediate HDL (28.8 ± 7.1 vs. 23.7 ± 5.8 mg/dL; *p* = 0.002) and large HDL (19.3 ± 11.1 vs. 10.7 ± 5.7 mg/dL; *p* = 0.005) was higher in females as compared to male patients whereas small HDL showed no gender differences (11.3 ± 3.6 vs. 11.3 ± 4.3 mg/dL; *p* = 0.99). Large HDL correlated inversely with weight (*r* = −0.28; *p* = 0.008) whereas the presence of diabetes, fasting glucose and HbA1c was not associated with HDL subfractions. Similarly, the presence of hypertension or smoking was not associated with HDL subfractions in our study population. HDL subfractions did also not differ in patients with high dose statin, low dose statin or without statin treatment (data not shown). In addition, the severity of disease, i.e. the number of diseased vessels was not associated small HDL (*p* = 0.22), intermediate HDL (*p* = 0.06) nor with large HDL (*p* = 0.29).

**Table 2:** Correlation of HDL subfractions and lipid parameters.

	Small HDL		Intermediate HDL		Large HDL	
	*R*	*p*-value	*R*	*p*-value	*R*	*p*-value
HDL	0.060	0.56	**0.68**	**<0.0001**	**0.80**	**<0.0001**
Total cholesterol	**0.39**	**<0.0005**	**0.60**	**<0.0001**	**0.24**	**0.023**
LDL	**0.30**	**<0.005**	**0.43**	**<0.0001**	0.11	0.30
VLDL	**0.42**	**<0.0001**	**0.22**	**0.042**	**−0.22**	**0.035**
Triglycerides	**0.36**	**<0.005**	0.02	0.86	**−0.37**	**<0.0005**
Small HDL	**–**		0.01	0.91	**−0.26**	**0.014**
Intermediate HDL	0.01	0.91	–		**0.63**	**<0.0001**
Large HDL	**−0.26**	**0.014**	**0.63**	**<0.0001**	**–**	

HDL high density lipoprotein; LDL low density lipoprotein; VLDL very low density lipoprotein; significant correlations are printed bold.

### 3.3 Monocyte subsets are associated with small HDL serum levels

**Fig. 2:**
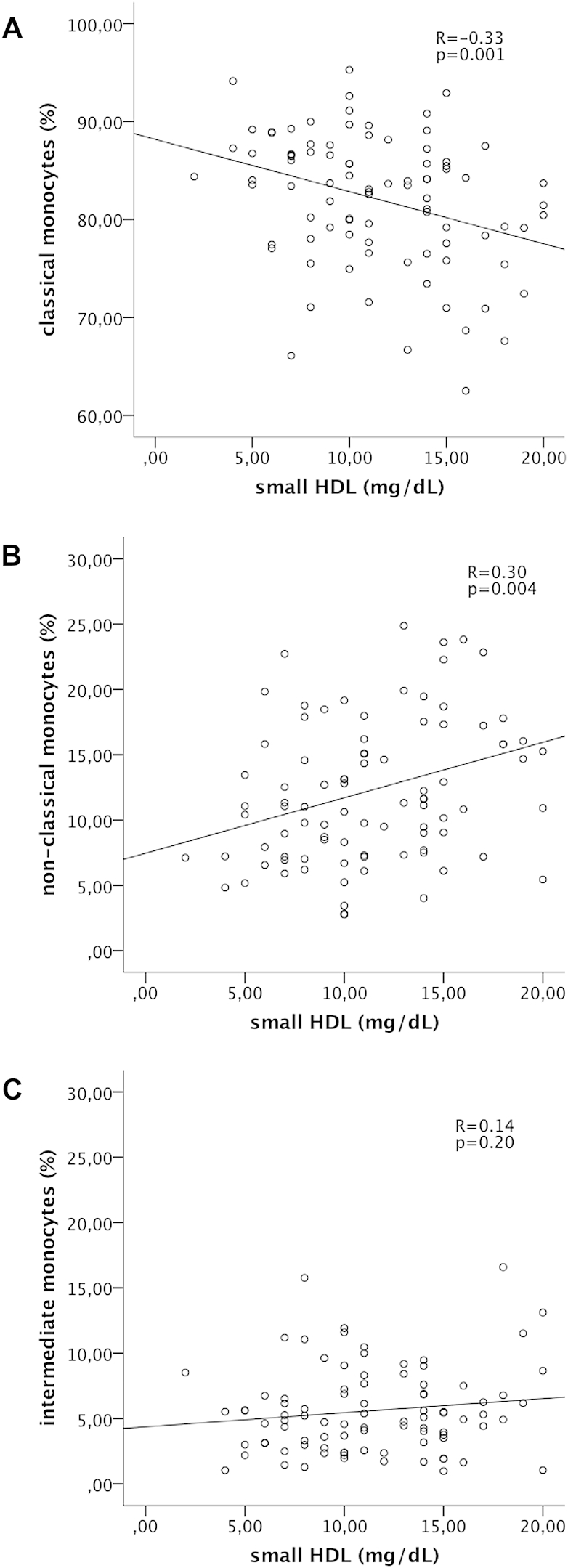
Correlation of small HDL serum levels and monocyte subsets. Given are scatter dot plots analyzing the association between small HDL levels and the proportion of classical monocytes (A), non-classical monocytes (B) and intermediate monocytes (C). *n* = 90.

**Fig. 3:**
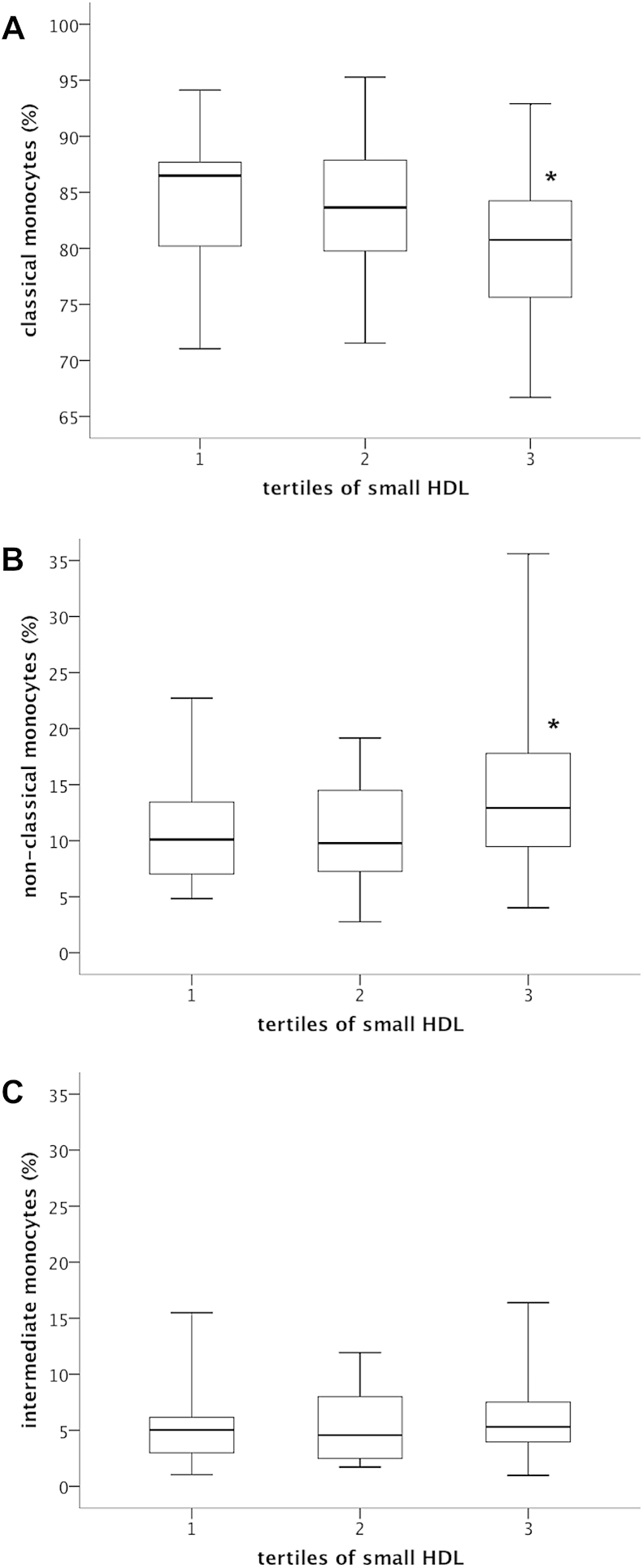
Monocyte subset distribution according to small HDL tertiles. Monocyte subset distribution is associated with small HDL serum levels. Boxplots indicate median, interquartile range (range from the 25th to the 75th percentile) and total range of classical monocytes (A), non-classical monocytes (B) and intermediate monocytes (C). *p < 0.01 for highest vs lower tertiles.

Monocyte subset distribution was determined by flow cytometry (Fig. 1). Mean number of CM was 270.5 ± 142.7 cells/μL (82.1 ± 6.7% of total monocytes), mean number of circulating NCM was 39.7 ± 28.9 cells/μL (12.3 ± 5.9% of total monocytes) and mean number of IM was 18.7 ± 15.1 cells/μL (5.6 ± 3.3% of total monocytes). Serum levels of small HDL showed an inverse relationship to the percentage of circulating CM (*r* = −0.33; *p* = 0.001; Fig. 2A) and correlated significantly with proportion of circulating pro-inflammatory NCM (*r* = 0.30; *p* = 0.004; Fig. 2B) while there was no association to IM (*r* = 0.14; *p* = 0.20; Fig. 2C). In contrast, intermediate HDL, large HDL and total HDL was not associated with monocyte subset distribution (Table 3). Linear regression analysis revealed that small HDL was associated with levels of pro-inflammatory NCM and circulating CM independently from other lipid parameters, risk factors and statin treatment regime. In contrast, IM were only associated with total and LDL cholesterol (Table 4). In particular, patients with small HDL levels in the highest tertile (13–20 mg/dL) showed the lowest levels of classical monocytes (79.3 ± 7% vs. 83.7 ± 6% and 83.9 ± 6%; *p* = 0.004; Fig. 3A) when compared with patients in the middle (9–12 mg/dL) and the lowest tertile (2–8 mg/dL). Additionally, patients in the highest tertile of small HDL exhibited the highest levels of pro-inflammatory NCM (14.7 ± 7% vs. 10.7 ± 5% and 10.8 ± 5%; *p* = 0.006; Fig. 3B), while levels of IM were not associated with tertiles of small HDL (5.9 ± 3% vs. 5.6 ± 3% vs. 5.3 ± 3%; *p* = 0.54; Fig. 3C).

**Table 3:** Correlation of HDL and HDL subfractions to circulating monocyte subsets.

Monocyte subsets	Classical monocytes CD14++CD16-		Intermediate monocytes CD14++CD16+		Non-classical monocytes CD14+CD16++	
	*R*	*p*-value	*R*	*p*-value	*R*	*p*-value
Total HDL	−0.08	0.45	−0.06	0.60	0.12	0.25
Small HDL	**−0.33**	**0.001**	0.14	0.20	**0.30**	**0.004**
Intermediate HDL	−0.05	0.66	−0.32	0.76	0.07	0.50
Large HDL	0.06	0.55	−0.12	0.26	−0.01	0.96

Significant correlations are printed bold.

**Table 4:** Multivariate regression models for the association of small HDL and circulating monocyte subsets.

	Classical monocytes CD14++CD16-			Intermediate monocytes CD14++CD16+			Non-classical monocytes CD14+CD16++		
	Univariate *p*-value	*β*	*p*-value	Univariate *p*-value	*β*	*p*-value	Univariate *p*-value	*β*	*p*-value
Small HDL	0.001	−0.33	0.006	0.20	0.21	0.08	0.004	0.27	0.02
Statin dose	0.21	0.14	0.19	0.45	−0.07	0.49	0.31	−0.12	0.25
Creatinine	0.36	−0.11	0.35	0.19	−0.18	0.12	0.07	0.23	0.05
VLDL	0.21	0.18	0.48	0.22	0.07	0.78	0.47	−0.25	0.32
Age	0.37	0.09	0.50	0.98	0.23	0.09	−0.23	−0.21	0.08
LDL	0.02	−0.19	0.56	0.05	0.80	0.01	0.11	−0.23	0.45
BMI	0.84	0.04	0.71	0.73	0.02	0.84	0.67	−0.06	0.58
Total cholesterol	0.02	−0.10	0.77	0.18	−0.86	0.01	0.06	0.60	0.08
Smoking	0.51	−0.03	0.78	0.02	0.32	0.006	0.61	−0.14	0.21
Hypertension	0.49	−0.03	0.81	0.12	0.16	0.15	0.94	−0.06	0.60
Log triglycerides	0.71	0.06	0.81	0.74	0.22	0.31	0.82	−0.19	0.39
Gender	0.96	0.01	0.99	0.12	0.14	0.28	0.42	−0.08	0.54

Total model *R*^2^		21%	0.040		24%	0.035		25%	0.031

HDL high density lipoprotein; Statin dose: High-dose statin treatment was defined as treatment with atorvastatin with a dosage of at least 40 mg or rosuvastatin at a dosage of at least 10 mg daily; VLDL very low density lipoprotein; LDL low density lipoprotein; BMI body mass index.

### 3.4 Association of HDL subfractions with circulating colony stimulating factors and inflammatory markers

Serum levels of small HDL correlated significantly with plasma levels of G-CSF (*r* = 0.22; *p* = 0.05) but not with plasma levels of GM-CSF (*r* = 0.05; *p* = 0.66) or M-CSF (*r* = −0.09; *p* = 0.37). Intermediate HDL, large HDL and total HDL were not associated with the three colony stimulating factors, respectively (data not shown). Serum levels of small HDL were not associated with the pro-inflammatory markers hsCRP (*r* = −0.05; *p* = 0.64) and IL-6 (*r* = −0.10; *p* = 0.38). In addition, the anti-inflammatory cytokine IL-10 was not associated with small HDL (*r* = 0.06; *p* = 0.62). Furthermore, intermediate HDL, large HDL and total HDL did not correlate with hsCRP, IL-6 and IL-10, respectively (data not shown). In addition, neither the pro-inflammatory markers hsCRP (CM: *R* = −0.11, *p* = 0.32; IM: *R* = 0.14, *p* = 0.19; NCM: *R* = 0.04, *p* = 0.68) or IL-6 (CM: *R* = 0.06, *p* = 0.59; IM: *R* = 0.13, *p* = 0.24; NCM: *R* = −0.14, *p* = 0.24) nor the anti-inflammatory marker IL-10 (CM: *R* = 0.01, *p* = 0.94; IM: *R* = −0.01, *p* = 0.98; NCM: *R* = −0.01, *p* = 0.94) were associated with circulating monocyte subsets.

## 4 Discussion

In the present study we are able to demonstrate that patients with stable coronary artery disease and elevated levels of small HDL exhibit a shift towards a pro-inflammatory monocyte subset distribution, indicated by elevated levels of non-classical monocytes (CD14+CD16++) and a reduced proportion of classical monocytes (CD14++CD16−). This was independent of established cardiovascular risk factors, such as BMI, presence of diabetes mellitus, smoking and plasma levels of hsCRP. Furthermore, neither serum lipoproteins nor statin treatment had an impact on the observed association of monocyte subsets and small HDL serum levels. Of note, intermediate monocytes (CD14++CD16+) were not associated with small HDL serum levels in the unadjusted analysis.

Large epidemiological and prospective population studies have clearly demonstrated an inverse correlation between serum HDL cholesterol levels and coronary heart disease risk [4]. Atheroprotective properties of HDL include cholesterol efflux, anti-oxidative, anti-inflammatory, cytoprotective, vasodilatatory and anti-thrombotic activities [32], [33]. Heterogeneity and varying functionality of HDL particles in different pathophysiological settings could make the HDL-hypothesis more complex.

Pre-clinical studies and studies involving HDL-subsets from healthy subjects provided evidence for potential anti-atherogenic effects of small HDL particles [8]. Interestingly, under dyslipidemic conditions, HDL metabolism and subfraction distribution is strongly altered to an increase in small HDL and a decrease of large HDL particles [28], [34], [35]. This was shown both in patients with merely elevated triglycerides but also in patients with elevated total cholesterol [36], [37]. Similarly to the above described findings, in our study population including 90 patients with angiographically proven stable coronary artery disease, small HDL serum levels were associated with an atherogenic lipid profile, characterized by an association with total cholesterol, LDL, VLDL as well as with triglyceride levels. On the other hand, there was no correlation between small HDL and total HDL levels to be found.

A great body of evidence suggests a shift towards small HDL levels not only in dyslipidemic and obese patients but also in patients with established cardiovascular disease. In 115 patients undergoing coronary angiography, the levels of large HDL were strongly increased in patients with established coronary artery disease [13]. In over 1000 adults followed for more than a decade, elevated levels of both small and large HDL levels were predictive for development of ischemic heart disease, however after adjustment small HDL levels lost their predictive power [38]. A reduced HDL particle size was further associated with presence of CAD in women and levels of small HDL were elevated in patients with acute ischemic stroke compared to healthy controls [39], [40]. In a carefully conducted study including 60 patients, small HDL levels were associated with non-calcified, larger and more unstable plaques as evidenced by coronary computer tomography and intravascular ultrasound [41]. A decreased HDL particle size was associated with an unfavorable cardiovascular risk profile and an increased risk for future CAD [28]. In a cohort of 102 patients suffering from myocardial infarction below the age of 40 and 200 matched controls, large and intermediate HDL was inversely correlated with premature AMI, while small HDL was higher in young AMI patients but statistical significance was lost after adjustment [30]. Patients with ACS exhibited elevated levels of small HDL and lower levels of large HDL when compared to patients with stable angina pectoris [42]. Furthermore, large HDL particles were inversely associated with disease severity and progression in 60 male myocardial infarction survivors below the age of 45 [14]. In contrast to the latter study, we were not able to demonstrate an association between HDL subfractions and severity of disease in our older study population.

Heterogeneity amongst monocytes, a cell population crucially involved in atherogenesis, has been demonstrated more than two decades ago by staining of the surface markers CD14 and CD16 [18]. CD16-positive monocytes were soon demonstrated to be expanded in patients with acute and chronic inflammatory diseases including atherosclerosis and these cells responded with a stronger activation upon inflammatory stimuli [20], [21], [43]. Subset specific interactions between lipoprotein metabolism and monocyte subset distribution have been suggested by various observational and interventional studies. A small cross-sectional study demonstrated an inverse correlation between NCM and HDL, while a following study from the same study group could not confirm this finding, however demonstrated a positive correlation between total cholesterol, LDL-cholesterol and triglycerides and the proportion of NCM [25], [26].

In our study, small HDL serum levels were inversely correlated with classical monocytes and showed a positive correlation with non-classical monocytes. In contrast to other studies, where the correlations between total HDL or LDL with monocyte subsets were diminished after adjustment for BMI [24], [44], the associations in our study were independent from traditional risk factors such as age, sex, current smoking, BMI and presence of diabetes, as well as statin treatment and lipoproteins in other models. When patients were stratified into three tertiles according to their small HDL levels, patients in the highest tertile showed a striking shift of monocyte subsets to a more atherogenic and pro-inflammatory distribution pattern, with an increased proportion of NCM and fewer CM. Interestingly, monocyte subsets did not correlate with plasma levels of the circulating inflammatory markers CRP, IL-6 or IL-10, respectively. Data on the relationship between monocyte subsets and circulating inflammatory markers is conflicting. While in a population of patients with unstable angina, CD16-positive monocytes were associated with hsCRP levels, another study from the same group demonstrated no relationship with hsCRP levels in patients with stable angina. Another study including a heterogeneous cohort of CAD-patients demonstrated an association of CD16-positive monocytes with TNF-α but not hsCRP or IL-6 [21], [22], [23]. Interestingly, a recently published study demonstrated an increased IL-6 receptor expression on CM and IM in patients with CAD as compared to a healthy control group [45]. In the same study, monocyte subsets derived from patients showed a lower response to stimulation with endotoxins when compared to monocytes derived from healthy individuals. In addition, small HDL levels did neither correlate with hsCRP or IL-6 nor with the anti-inflammatory marker IL-10. Therefore one could speculate that it is more likely that small HDL may have direct cellular effects and not distinct systemic pro-inflammatory properties. The family of colony-stimulating factors including M-CSF, G-CSF and GM-CSF were first characterized by their hematopoietic abilities. Today it is accepted that many vascular cells can express and react to CSFs and that CSFs are involved in atherogenesis via modulation of macrophage phenotype altering their inflammatory potential and cholesterol uptake abilities [46]. Interestingly, in cardiovascular disease, only for M-CSF plasma levels a prognostic value was described [47]. In our study, only plasma levels of G-CSF, the CSF least characterized within atherogenesis, showed a weak, but statistically significant association with small HDL levels.

As this is a cross-sectional study, we can only analyze associations between HDL subfractions and monocyte subsets at one time point and cannot draw any functional conclusions of these associations during atherogenesis. In addition, it has to be emphasized that this study was performed in patients with established CAD. Therefore it is not possible to draw any conclusions for healthy subjects. Whether small HDL has dysfunctional anti-inflammatory or even pro-inflammatory properties in patients with established disease or whether an increase of small HDL is merely a marker for a pro-atherogenic and pro-inflammatory environment has to be investigated in further studies.

In conclusion, we demonstrate evidence for an association between small HDL levels and an increase of the non-classical monocyte subset population accompanied by a decrease in the proportion of classical monocytes. Our findings demonstrate a pro-inflammatory association between small HDL and innate immunity in the setting of stable atherosclerotic disease.

## Conflict of interest

The authors declare no potential conflicts of interest.
